# An Efficient and Effective Deep Learning-Based Model for Real-Time Face Mask Detection

**DOI:** 10.3390/s22072602

**Published:** 2022-03-29

**Authors:** Shabana Habib, Majed Alsanea, Mohammed Aloraini, Hazim Saleh Al-Rawashdeh, Muhammad Islam, Sheroz Khan

**Affiliations:** 1 Department of Information Technology, College of Computer, Qassim University, Buraydah 52571, Saudi Arabia; s.habibullah@qu.edu.sa; 2 Computing Department, Arabeast Colleges, Riyadh 13544, Saudi Arabia; 3 Department of Electrical Engineering, College of Engineering, Qassim University, Qassim 52571, Saudi Arabia; mo.aloraini@qu.edu.sa; 4 Cyber Security Department, College of Engineering and Information Technology, Onaizah Colleges, Onaizah 56447, Saudi Arabia; hazim@oc.edu.sa; 5 Department of Electrical Engineering, College of Engineering and Information Technology, Onaizah Colleges, Onaizah 56447, Saudi Arabia; m.islam@oc.edu.sa (M.I.); sheroz@oc.edu.sa (S.K.)

**Keywords:** COVID-19, convolution neural network, data augmentation, deep learning, face mask, machine learning, classification, MobileNet, autoencoder

## Abstract

Since December 2019, the COVID-19 pandemic has led to a dramatic loss of human lives and caused severe economic crises worldwide. COVID-19 virus transmission generally occurs through a small respiratory droplet ejected from the mouth or nose of an infected person to another person. To reduce and prevent the spread of COVID-19 transmission, the World Health Organization (WHO) advises the public to wear face masks as one of the most practical and effective prevention methods. Early face mask detection is very important to prevent the spread of COVID-19. For this purpose, we investigate several deep learning-based architectures such as VGG16, VGG19, InceptionV3, ResNet-101, ResNet-50, EfficientNet, MobileNetV1, and MobileNetV2. After these experiments, we propose an efficient and effective model for face mask detection with the potential to be deployable over edge devices. Our proposed model is based on MobileNetV2 architecture that extracts salient features from the input data that are then passed to an autoencoder to form more abstract representations prior to the classification layer. The proposed model also adopts extensive data augmentation techniques (e.g., rotation, flip, Gaussian blur, sharping, emboss, skew, and shear) to increase the number of samples for effective training. The performance of our proposed model is evaluated on three publicly available datasets and achieved the highest performance as compared to other state-of-the-art models.

## 1. Introduction

The face mask-wearing trend in public is growing all over the world due to COVID-19. Before COVID-19 the community wore masks to protect themselves from air pollution, while some people in the community used them because of self-consciousness regarding their looks [1]. Currently, scientists and domain experts confirm that wearing a face mask during this pandemic reduce the transmission of COVID-19 [2]. Coronavirus, also known as COVID-19, or the most recent epidemic virus, hit humans around the end of the year 2019 [3]. The rapid global spread of this disease forced the WHO to declare it a global pandemic. As stated by [4], COVID-19 infected more than five million people throughout 188 countries within just six months, and currently, the number of people infected has increased substantially. The COVID-19 virus transfers from one person to another through close contact in crowded areas or through the sharing of multiple gadgets in a public environment, as well as in indoor environments such as hotels, cafes, etc.

The COVID-19 pandemic has given rise to an extraordinary degree of worldwide scientific cooperation. Machine learning and deep learning based algorithms are very helpful in the fight against COVID-19 in many aspects [5]. These algorithms also allow the research community and clinicians a vast quantity of data evaluation for COVID-19 distribution forecasting. It serves as an initial warning technique for possible pandemics and to classify the population according to vulnerably. Healthcare organizations are in need of funding for advancing technologies with the help of the Internet of things, big data, and artificial intelligence, which will help to predict and tackle new diseases due in the aftermath of this pandemic. Artificial intelligence-based algorithms are explored to detect infection rates [6], to detect the presence of COVID-19 using chest X-ray images [7,8], and to detect and monitor social distancing [9], the wearing of face masks, etc.

Policymakers are facing several risks and challenges in reducing the spreading of COVID-19 and managing its effects [10]. To avoid and prevent the spread of COVID-19, all countries have adopted several rules such as a stay-at-home policies [11], social distancing [9], city lockdowns [12], travel bans [13], requiring the wearing of face masks in public areas, etc. These government regulations are deployed as actions to reduce the transmission of the pandemic. However, the monitoring process of a large group of people or crowded area is very difficult using manual monitoring systems. To overcome such problems, the introduction of efficient and effective face mask detection systems is required.

In the light of literature, the researchers are mainly focused on the current challenges related to COVID-19, such as social distancing [14], face mask detection [15], COVID-19 detection using chest x-ray images [16], etc. Face mask detection is one of the challenging areas for the research community. Regarding creating methods of face mask detection, some attempts have already been made, as mentioned in the recent literature. For instance, Qin et al. [17] developed a method to identify different conditions of wearing a face mask such as a face without mask, correctly wearing a face mask, and incorrectly wearing a face mask. In this work, the authors developed a hybrid network with the combination of image super-resolution and classification networks. Their proposed method includes four main steps i.e., preprocessing, face detection, image super-resolution, and face mask condition identification. Ejaz et al. [18] developed a principal component analysis-based model for person identification through a face mask and with no mask detection. In the literature of face detection models, this model achieved state-of-the-art accuracy, where the detailed reviews are found in [19,20,21]. Ejaz et al. [18] claim that the accuracy of face detection models is dropped below 70% when it recognizes the face while wearing a mask. To remove mask objects from the face, Din et al. [22] present a novel technique by utilizing the generative adversarial network. Their proposed model includes two discriminators: the first discriminator is used to extract the global face mask structure, and the second discriminator is used to extract the face mask missing region. They evaluated their model using a paired synthetic dataset and achieved high accuracy in the removal of the face masks. GE et al. [23] collected a dataset and developed a deep learning-based model to recognize normal and face masks in the general population. Their proposed model is based on Convolutional Neural Network (CNN) architecture that includes the proposal module, the embedding module, and the verification module. To classify face masks, Loey et al. [1] developed a hybrid model with a combination of CNN and machine learning techniques. The CNN models are used to extract important features from the face mask and face unmask image, followed by the use of a decision tree, support vector machine, and ensemble classifiers. The combination of several models makes it computationally expensive, requiring powerful GPUs and TPUs for their execution. Furthermore, Teboulbi et al. [24] developed a deep-learning based model for face mask detection and social distancing measurement by utilizing different CNN-based architectures. In short, several articles presented in the recent literature for face mask detection are based on CNN architectures [25,26,27]. In these articles, the authors compared the performance of two or three CNN-based architectures and proposed a model which achieved comparatively high accuracy. However, comparison of two or three models is not sufficient for an in-depth analysis of face mask detection considering the accuracy and running time. Furthermore, the current models developed for face mask detection has lower accuracy and are computationally expensive. To reduce the transmission rate of COVID-19, early face mask detection, with high accuracy and lower computational complexity, is very important to ensure its implementation on resource-constrained devices. Therefore, in this work, we investigate several lightweight models for face mask detection. After a set of extensive experiments, we introduced a lightweight, deep learning-based model based on a MobileNet architecture for face mask detection. The proposed model utilizes MobileNet as a backbone architecture, used to extract meaningful information from the input data, followed by encoding layers to squeeze the information for effective training. The main contributions of the proposed work are as follows:

- For the sake of face mask detection, a limited number of datasets are available with a limited number of images. Therefore, we applied extensive data augmentation techniques to increase the number of samples for effective training and validation output.
- We developed an efficient and effective model for face mask detection. The proposed model is based on MobileNet architecture, followed by an autoencoder to select the best optimal feature for final classification. The proposed model is developed after extensive experiments over several deep learning-based models with different parameters.
- The performance of several models is evaluated in this work using benchmark datasets, and the proposed model achieved the highest accuracy rate as compared to the state-of-the-art models. Furthermore, the efficiency of the proposed model is also evaluated on edge devices to ensure their implementation in real-world scenarios.

The balance of the paper is organized as follows: Section 2 briefly describes the proposed model. The experimental results and comparison with other state-of-the-art models are presented in Section 3, and finally, Section 4 concludes the manuscript.

## 2. Proposed Model

**Figure 1:**
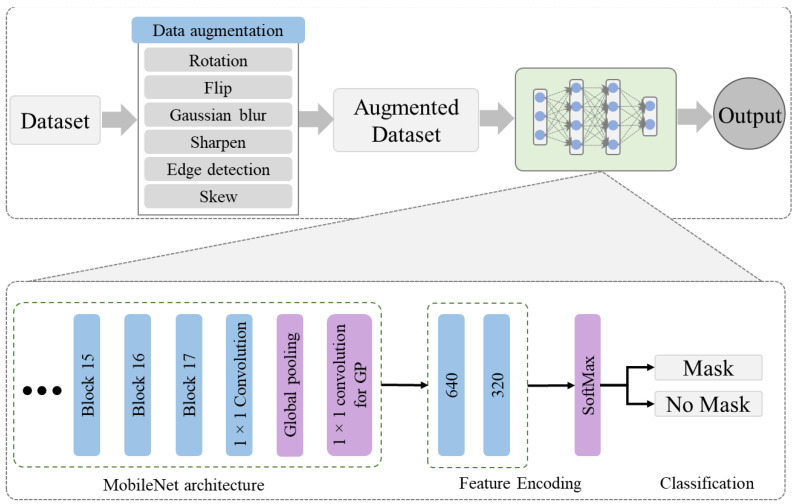
The visual representation of the proposed model.

In this work, we developed an effective and efficient model for face mask detection based on the Convolutional Neural Network (CNN). Motivated by the high performance of CNN in several domains such as video analysis [28], classification [29], time-series data analysis [30], electricity prediction [31], and many others, in this work, we developed a CNN-based model for face mask detection. The visual representation of the proposed work is given in Figure 1, which includes two main phases of data augmentation and the proposed model. These phases of the proposed work are briefly described in the following subsequent sections.

### 2.1. Data Augmentation

The data augmentation process is briefly described in this section. Abundant and high-quality data is the main requirement for the effective training of deep learning models [32]. The proposed model for face mask detection is evaluated using the different datasets, as mentioned in Section 3, where these datasets have a limited number of training samples and the deep learning-based models require a large amount of data for effective training. Thus, to achieve high accuracy and increase the number of samples in the datasets for effective deployment of the model, we applied several data augmentation techniques to increase the number of samples in the datasets. The details about data augmentation techniques and their corresponding values are given in Table 1. These techniques include flipping, rotation, shearing, skewing, sharpening, emboss, and blurring. We include a total number of 7 techniques and 20 parameters. By utilizing these techniques, we increase the number of samples in the datasets to achieve high accuracy for face mask detection. Each value of the parameters is selected based on the nature of the data, for example, the possible degree of face rotation in the general scenario is between −15 and 15, where the details are given in [33]; another possible rotation for faces is right and left flipping, while the other parameters such as Gaussian blur, sharpness, shear, etc. are initialized based on the nature of the data.

### 2.2. Backbone Architecture

In this section, we briefly describe the internal architecture of the proposed model for face mask detection. Before selecting the proposed model, we conducted an extensive ablation study to select the best optimal model for face mask detection. We perform experiments on different deep learning-based architectures such as VGG16, VGG19, InceptionV3, NasNetMobile, MobileNetV1, MobileNetV2, ResNet-101, ResNet-50, EfficientNet, and the proposed MobileNetV2 autoencoder model. These models are tested with several sets of configurations, such as a number of epochs, learning rate, etc., to improve the detection accuracy and develop an appropriate model for face mask detection. After a detailed ablation study as given in the results section, we found that MobileNetV2 provides high accuracy as compared to other models, and this model is also computationally inexpensive. The main blocks of the MobileNetV2 architecture are the residual connection in the bottlenecks. These bottlenecks with residual connection included convolutional blocks, where the start and end of each convolutional block are connected with each other through a skip connection mechanism. Based on the skip connection mechanism, the MobileNetV2 can retrieve earlier activations that are not updated in each convolutional block. The internal architecture of MobileNetV2 includes a convolutional layer, followed by residual bottlenecks. A total number of 19 residual blocks are used in MobileNetV2 architecture. Further convolutional and pooling layers are incorporated with MobileNetV2 architecture after the bottlenecks. The detail about the internal architecture of MobileNetV2 is given in Table 2. This architecture is trained on the ImageNet dataset, which includes 1000 classes. We finetuned the internal architecture of MobileNetV2 and used it as the backbone architecture in the proposed model.

### 2.3. Proposed Architecture

**Figure 2:**
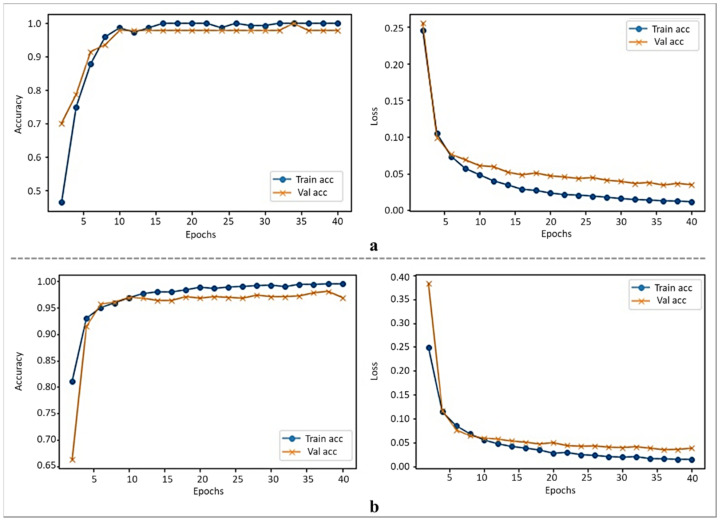
The training loss and accuracy of the proposed model, (**a**) the loss and accuracy over the FMD dataset; (**b**) the loss and accuracy over the FM dataset.

In this work, we used MobileNetV2 architecture, followed by autoencoders. The MobileNetV2 is an efficient and effective deep learning-based architecture among several available choices, i.e., VGG16, AlexNet, EfficientNet, etc. In the proposed model, MobileNetV2 is used as the backbone architecture for features extraction, followed by an encoded layer to select optimal features. The autoencoder includes two main models, an encoder and decoder, which are commonly used for unlabeled data. The encoder is used to encode the input feature map, followed by a decoder module to reconstruct the feature map. In this work, we utilized the encoder module of the autoencoder to squeeze the output feature vector from the MobileNetV2 architecture for a more abstract representation of the features. The output dimensions of the MobileNetV2 architecture are 7 × 7 × 1280, which are reduced to 1280 dimensions by applying global average pooling. The output of the global average pooling is then forwarded to the proposed encoding mechanism to further extract more representative features for final classification. The 1280 dimensions of the features vector are first encoded to 640 dimensions, and then 320 dimensions. The main reason behind the feature encoding using their halves is to reduce the complexity of the autoencoder [34]. In this work, we used stacked encoding layers to transform the high dimensional output feature vector of MobileNetV2 into low dimensions, with an abstract representation of all features maps. In the encoding module of the autoencoder, the weights are multiplied with the data, including a bias term and an activation function such as ReLU or Sigmoid. In the proposed stacked encoded layers, the first encoding layer takes the output feature vector of MobileNetV2, while the second layer uses previous layer features in a stacked mechanism. The output of the encoding layers is then forwarded to two fully connected (Dense) layers to learn the encoded features prior to the classification layer. The proposed architecture is developed after extensive experiments over different combinations of encoding layers, finally achieving the highest performance with the aforementioned configuration. The internal architecture of the proposed model, such as layers information, the output shape of each layer, and their parameters, are given in Table 3. The proposed model is trained for 40 epochs, and the training loss and accuracy graphs over both datasets are given in Figure 2.

## 3. Results and Discussion

In this section, the experimental results are described in detail. The performance of several models is tested before selecting the proposed model. All the experiments are carried out on GeForce RTX 2070 GPU, with 8 GB memory using the Keras framework with backend TensorFlow. This section describes the datasets used for the evaluation of each model, evaluation metrics, a detailed ablation study, and a comparison with state-of-the-art models developed for face mask detection. Furthermore, the time complexity of the proposed model is also tested using several hardware specifications such as GPU, CPU, and edge devices. All these sections are briefly described in the subsequent sections.

### 3.1. Evaluation Metrics

For performance evaluation, we used several evaluation metrics such as accuracy, precision, recall, False Positive (FP), False Negative (FN), True Positive (TP), True Negative (TN), and F1-scores. Accuracy is a metric used in classification tasks to evaluate model performance and how the model performs among all the classes. The mathematical representation of accuracy is given in Equation (1). Precision is the ratio between the number of samples classified as positive and all samples where the mathematical representation is given in Equation (2). The recall is the ratio between positive samples classified as positive and the total number of samples as shown in Equation (3). The F1-score is the harmonic mean of recall and precision. The mathematics behind the F1-score are given in Equation (4).

(1) $$\mathrm{Accuracy}\;=\frac{\mathrm{TP}\;+\;\mathrm{TN}}{\mathrm{TP}\;+\;\mathrm{FN}\;+\;\mathrm{TN}\;+\;\mathrm{FP}}$$

(2) $$\mathrm{Precision}\;=\frac{\mathrm{TP}}{\mathrm{TP}\;+\;\mathrm{FP}}$$

(3) $$\mathrm{Recall}\;=\frac{\mathrm{TP}}{\mathrm{TP}\;+\;\mathrm{FN}}$$

(4) $$F1-\mathrm{score}=2\;\cdot \;\frac{\mathrm{Precision}\;\times \;\mathrm{Recall}}{\mathrm{Precision}\;+\;\mathrm{Recall}}\;$$

### 3.2. Datasets

In this work, we used three datasets as Face Mask Detection (FMD) [35], Face Mask (FM) [36], and Real-World Mask Face Recognition (RMFR). In the FMD dataset, there is a total number of 7553 images in which 3725 images belong to the face mask while the remaining images are from the without face mask class. In this dataset, around 700 images simulate face mask images while the remaining show real-world face mask images. In the FM dataset, there are a total number of 1376 images, of which 690 images belong to the face mask class, while the rest belong to the without face mask class. The RMFR dataset includes 5000 face mask images and 90,000 images without masks. There is a limited number of samples in two datasets, and the deep learning-based models require a huge amount of data for effective training. Considering the limited numbers of samples in this work, we apply extensive data augmentation techniques to increase the number of samples in each dataset. The RMFR dataset includes a huge number of samples without masks; however, deep learning-based models require a balanced amount of data for effective training. Therefore, we balance the dataset before training the model. Table 4 represents the number of samples in the original dataset and the augmented dataset.

### 3.3. Ablation Study

**Figure 3:**
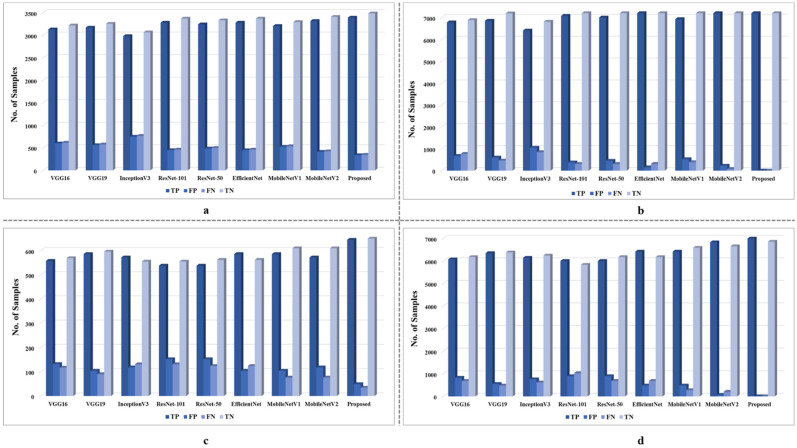
The detailed performance of each model in terms of TP, TN, FP, and FN where (**a**)—FMD original dataset, (**b**)—FMD augmented dataset, (**c**) FM—original dataset, and (**d**)—FM augmented dataset.

**Figure 4:**
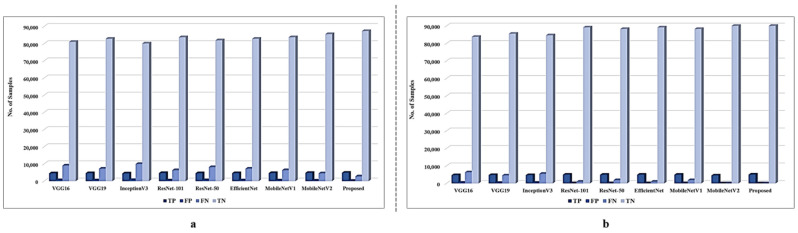
The detailed performance of each model in terms of TP, TN, FP, and FN over RMFR where (**a**)—original dataset, (**b**)—balanced dataset.

Before selecting the proposed model, the extensive ablation study of the deep learning-based models is conducted to develop an efficient and effective model for face mask detection. These models include VGG16 [37], VGG19 [37], InceptionV3 [38], NasNetMobile [39], MobileNetV1 [40], MobileNetV2 [41], ResNet-101 [42], ResNet-50, EfficientNet [43], and the proposed MobileNetV2 autoencoder model. The performance of these models is evaluated on three benchmark datasets. The performance of each model in terms of TP, TN, FP, and FN is given in Figure 3 and Figure 4, whereas the detailed performance of the proposed and other models in terms of accuracy, precision, recall, and F1-score are given in Table 5 and Table 6.

The performance of each model is lower in terms of accuracy over the original dataset as compared to the augmented and unbalanced dataset. In an overall comparison, the proposed model achieved the highest precision, recall, F1-score, and accuracy in both scenarios over all datasets. For instance, the proposed model achieved 0.9098, 0.9076, 0.9087, and 0.9098 precision, recall, F1-score, and accuracy, respectively, over the original FMD dataset, while these values are 0.9997, 1.0, 0.9999, and 0.9999, respectively, over the FMD augmented dataset. For the original FM dataset, the proposed model achieved 0.9348, 0.9499, 0.9423, and 0.9426 precision, recall, F1-score, and accuracy, respectively, and 0.9993, 0.9994, 0.9994, and 0.9994 precision, recall, F1-score, and accuracy, respectively. Compared to other methods the proposed model achieved better accuracy for ensuring its implementation for face mask detection. Comparatively, the second-highest performance is achieved by MobileNetV2 in terms of accuracy, precision, recall, and F1-score. For instance, MobileNetV2 achieved 0.8792 precision, 0.8948 recall, 0.8869 F1-score, and 0.8894 accuracy over the original FMD dataset, while it achieved 0.9699, 0.9895, 0.9796, and 0.9801 precision, recall, F1-score, accuracy, respectively, over the augmented FMD dataset. Similarly, MobileNetV2 also achieved the second-highest performance of the FD original and the FD augmented dataset, where the details are given in Figure 3, and Table 5. Furthermore, the proposed model also achieved the highest performance over the RMFR dataset, and the detailed results over the balanced and unbalanced data is given in Figure 4 and Table 6. For instance, the proposed model achieved 0.9498, 0.5134, 0.6665, and 0.9516 precision, recall, F1-score, and accuracy, respectively, over the unbalanced RMFR dataset, while these values are 0.9998, 0.9998, 0.9998, and 1, respectively, over the RMFR balanced dataset.

### 3.4. Comparison with Baselines

In the literature, some studies have been done for face mask detection technology. However, the detection accuracy needs to be improved to protect the transmission of COVID-19. In the light of the literature, several detection methodologies are developed to recognize faces with masks and faces without masks. In this section, we compare the performance of the proposed model with other models. For instance, the performance of our model is compared with Militante et al. [44], Chen et al. [45], Hariri et al. [46], Oumina et al. [36], and Loey et al. [1]. Militante et al. [44] developed a deep learning-based model for face mask detection and achieved 0.975 precision, 0.945 recall, 0.955 F1-score, and 0.96 accuracy. Chen et al. [45] achieved 0.9480 accuracy, Hariri et al. [46] achieved 0.913 accuracy, and Oumina et al. [36] achieved 0.9184 precision, 0.9508 recall, and 0.9711 accuracy. The average precision, recall, F1-score, and accuracy results of Loey et al. [1] are 97.4, 97.3, 97.3, and 97.4, respectively. Compared to these studies, on average, the proposed model achieved 0.9996 precision, 0.9997 recall, 0.9997 F1-score, and 0.9998 accuracy. A detailed comparative analysis of the above-mentioned models with the proposed model is shown in Table 7.

### 3.5. Evaluation Using Edge Devices

The current surveillance systems have limited computational capabilities and cannot run deep learning-based computationally expensive models. For this purpose, the researchers and domain experts transmit these videos to the cloud or local servers to process them and then extract meaningful information such as face mask detection. The transmission of data to these servers utilizes a huge amount of bandwidth, sometimes causing a delay, and these servers are costly. Besides this, the processing of surveillance data over edge devices is very important for providing fast and inexpensive processing. However, the current surveillance sensors have limited memory and processing capabilities; therefore, in this work, we used resource-constrained devices to process these videos for efficient face mask detection. For this purpose, we evaluated the efficiency of the proposed model using three types of settings as a resource-constrained device (Raspberry Pi), a CPU, and a GPU with an input size of 224 × 224 × 3. The details regarding the hardware specifications of each device are given in Table 8. The time complexity of the proposed model is evaluated on Frame Per Second (FPS), which shows how many samples the proposed model processes in a second. The lightweight architecture of the proposed model achieved 199.01 FPS over GPU, 44.06 FPS over CPU, and 18.07 FPS over the Raspberry Pi resource-constrained device. The FPS of the proposed model over the resource-constrained device is lower than over the other devices; however, the processing of a model with 18.07 FPS is enough for the real-time implementation of a system that ensures its adaptability over edge devices.

## 4. Conclusions

Due to the COVID-19 pandemic, each country in the world is facing huge health crises and the governments are struggling to control and prevent the transmission of the Coronavirus. In the light of literature, wearing a face mask is the most efficient way to control the spread of the virus. Governments have instituted the mandatory wearing of face masks in public areas, which is difficult to monitor manually. Therefore, in this work, we developed an automatic face mask detection model with high accuracy that is also computationally inexpensive. The proposed model is based on the use of MobileNet, followed by an autoencoder. The MobileNet architecture is used to extract meaningful features from the input data, which are then forwarded to the encoding layers to select the optimal features. These optimal features are then used for the final classification. The performance of the proposed model is evaluated on benchmark datasets, and the results reveal significant improvements in accuracy, ensuring the implementation of the proposed model for face mask detection. Furthermore, the performance of the proposed model is also evaluated on resource-constrained devices to ensure their implementation over edge devices. The proposed model achieved the highest accuracy and the lowest running time as compared to other state-of-the-art techniques. In the future, we will extend this work to include the positioning of face masks, such as a face with no mask, a face with a mask, and a face with an incorrect mask. For this purpose, we will investigate emerging technologies such as explainable artificial intelligence, reinforcement learning, active learning, and lifelong learning techniques for face mask positioning and detection.

## Figures and Tables

**Table 1:** The data augmentation with a range of parameters.

S. No	Technique	Parameter
1	Rotation (degree angle)	−15–15
2	Flip	Right, left
3	Gaussian Blur (value of sigma)	0.25, 0.50, 0.75, 1.0
4	Sharping (value of lightness)	0.50, 1.00, 1.50, 2.00
5	Emboss (value of strength)	0.50, 1.00, 1.50, 2.0
6	Skew (Tilt)	Right, left
7	Shear	x-axis and y-axis, 10 degrees

**Table 2:** The internal architecture of MobileNetV2.

Layer	Repetition	Size of Stride
Convolution 3 × 3	1	2
Bottleneck	1	1
	2	2
	3	2
	4	2
	3	1
	3	2
	1	1
Convolution 1 × 1	1	1
Pooling 7 × 7	1	-
Convolution 1 × 1	1	-

**Table 3:** The internal architecture of the proposed model.

Type of Layer	Output Shape	Params.
MobileNetV2	7 × 7 × 1280	2,257,984
Global average pooling	1280	-
Encoder1	640	819,840
Encoder1	320	205,120
Dense	64	20,544
Dense	32	2080
Dense	2	66
**Total params.**		**3,305,634**

**Table 4:** The number of samples in the original and augmented datasets.

Dataset	Original		Augmented	
	Mask	Normal	Mask	Normal
FMD	3725	3828	7450	7656
FM	690	686	6900	6860

**Table 5:** The detailed comparative analysis of different models for face mask detection.

Dataset	Data Type	Model	Precision	Recall	F1-Score	Accuracy
FMD	Original data	VGG16	0.8295	0.8431	0.8363	0.8397
		VGG19	0.8389	0.8527	0.8457	0.849
		InceptionV3	0.7893	0.8011	0.7951	0.7993
		ResNet-101	0.8698	0.884	0.8769	0.8795
		ResNet-50	0.8792	0.8585	0.8687	0.8689
		EfficientNet	0.8094	0.9178	0.8602	0.8702
		MobileNetV1	0.8698	0.8493	0.8594	0.8596
		MobileNetV2	0.8792	0.8948	0.8869	0.8894
		**Proposed**	**0.9098**	**0.9076**	**0.9087**	**0.9098**
	Augmented data	VGG16	0.9099	0.8985	0.9042	0.9048
		VGG19	0.9199	0.9371	0.9284	0.93
		InceptionV3	0.8599	0.8838	0.8717	0.8751
		ResNet-101	0.9499	0.9584	0.9542	0.955
		ResNet-50	0.9399	0.958	0.9488	0.95
		EfficientNet	0.9799	0.9596	0.9696	0.9697
		MobileNetV1	0.9299	0.9476	0.9387	0.9401
		MobileNetV2	0.9699	0.9895	0.9796	0.9801
		**Proposed**	**0.9997**	**1.0**	**0.9999**	**0.9999**
FM	Original data	VGG16	0.8087	0.8267	0.8176	0.819
		VGG19	0.8493	0.8669	0.858	0.859
		InceptionV3	0.829	0.8137	0.8212	0.819
		ResNet-101	0.7797	0.8042	0.7918	0.7943
		ResNet-50	0.7797	0.8127	0.7959	0.7994
		EfficientNet	0.8493	0.8254	0.8371	0.8343
		MobileNetV1	0.829	0.8827	0.855	0.859
		MobileNetV2	0.8493	0.8852	0.8669	0.8692
		**Proposed**	**0.9348**	**0.9499**	**0.9423**	**0.9426**
	Augmented data	VGG16	0.8799	0.8983	0.889	0.8898
		VGG19	0.9199	0.9296	0.9247	0.9249
		InceptionV3	0.8899	0.9086	0.8991	0.8999
		ResNet-101	0.8699	0.8535	0.8616	0.8599
		ResNet-50	0.8699	0.8973	0.8834	0.8848
		EfficientNet	0.9299	0.9033	0.9164	0.9149
		MobileNetV1	0.9299	0.9589	0.9442	0.9448
		MobileNetV2	0.9899	0.9707	0.9802	0.9799
		**Proposed**	**0.9993**	**0.9994**	**0.9994**	**0.9994**

**Table 6:** The detailed comparative analysis of different models over the RMFR balanced and unbalanced dataset.

Data Type	Model	Precision	Recall	F1-Score	Accuracy
Original data	VGG16	0.8798	0.2894	0.4355	0.8884
	VGG19	0.8998	0.3333	0.4864	0.9071
	InceptionV3	0.9198	0.3897	0.5475	0.9221
	ResNet-101	0.8898	0.31	0.4598	0.8965
	ResNet-50	0.9298	0.4246	0.5829	0.9394
	EfficientNet	0.9098	0.3596	0.5155	0.9173
	MobileNetV1	0.9198	0.3897	0.5475	0.9245
	MobileNetV2	0.9298	0.4246	0.5829	0.9328
	**Proposed**	**0.9498**	**0.5134**	**0.6665**	**0.9516**
Balanced data	VGG16	0.9298	0.4246	0.5829	0.9334
	VGG19	0.9498	0.5134	0.6665	0.9529
	InceptionV3	0.9398	0.4652	0.6224	0.9422
	ResNet-101	0.9898	0.846	0.9123	0.9934
	ResNet-50	0.9798	0.7312	0.8374	0.9881
	EfficientNet	0.9898	0.846	0.9123	0.9935
	MobileNetV1	0.9798	0.7312	0.8374	0.9874
	MobileNetV2	0.9993	0.9973	0.9983	0.9998
	**Proposed**	**0.9998**	**0.9998**	**0.9998**	**1**

**Table 7:** A comparative analysis of the proposed model with other state-of-the-art models.

Model	Precision	Recall	F1-Score	Accuracy
Militante et al. [44]	0.975	0.945	0.955	0.96
Chen et al. [45]	-	-	-	0.9480
Hariri et al. [46]	-	-	-	0.913
Oumina et al. [36]	0.9484	0.9508	-	0.9711
Loey et al. [1]	0.9963	0.9963	0.9945	0.9964
**Proposed**	**0.9996**	**0.9997**	**0.9997**	**0.9998**

**Table 8:** The hardware specification of each setting.

Setting	Memory	Model
Raspberry Pi	4 GB	Raspberry Pi 4 B+
CPU	32 GB	AMD Ryzen 5 5600X 6-Core Processor
GPU	8 GB	RTX 2070
